# A nnU-Net-based automatic segmentation of FCD type II lesions in 3D FLAIR MRI images

**DOI:** 10.3389/frai.2025.1601815

**Published:** 2025-06-27

**Authors:** Shubham Joshi, Millie Pant, Arnav Malhotra, Kusum Deep, Vaclav Snasel

**Affiliations:** ^1^Department of Applied Mathematics and Scientific Computing, IIT Roorkee, Roorkee, India; ^2^Mehta Family School of Data Science and Artificial Intelligence, IIT Roorkee, Roorkee, India; ^3^Lassonde School of Engineering, York University, Toronto, ON, Canada; ^4^Department of Mathematics, IIT Roorkee, Roorkee, India; ^5^Department of Computer Science, VŠB-Technical University of Ostrava, Ostrava, Czechia

**Keywords:** epilepsy, focal cortical dysplasia, segmentation, nnU-Net, deep learning

## Abstract

Focal cortical dysplasia (FCD) type II is a common cause of epilepsy and is challenging to detect due to its similarities with other brain conditions. Finding these lesions accurately is essential for successful surgery and seizure control. Manual detection is slow and challenging because the MRI features are subtle. Deep learning, especially convolutional neural networks, has shown great potential in automating image classification and segmentation by learning and extracting features. The nnU-Net framework is known for its ability to adapt its settings, including preprocessing, network design, training, and post-processing, to any new medical imaging task. This study employs an automated slice selection approach that ranks axial FLAIR slices by their peak voxel intensity and retains the five highest-ranked slices per scan, thereby focusing the network on lesion-rich slices and uses nnU-Net to automate the segmentation of FCD type II lesions on 3D FLAIR MRI images. The study was conducted on 85 FCD type II subjects and results are evaluated through 5-fold cross-validation. Using nnU-Net’s flexible and robust design, this study aims to improve the accuracy and speed of lesion detection, helping with better presurgical evaluations and outcomes for epilepsy patients.

## 1 Introduction

**Figure 1:**
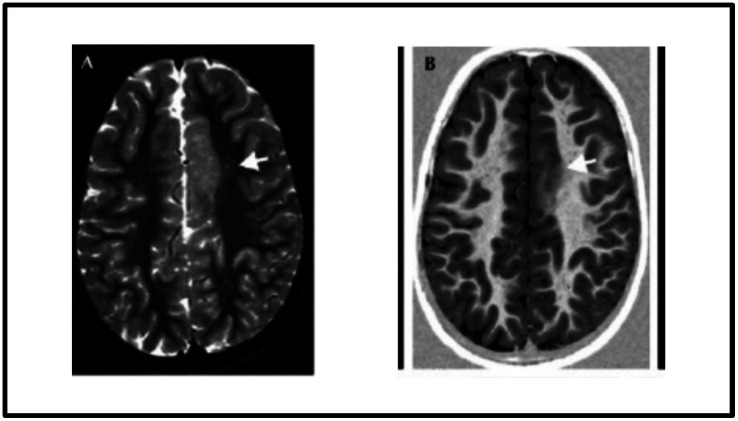
MRI images of FCD type II.

Focal cortical dysplasia (FCD) is one of the major causes of epilepsy that poses a significant challenge to detect due to its type, location and characteristics overlapping with other neurological conditions such as low-grade tumours and different kinds of cortical abnormalities. The surgical resection of the epileptic zones in the FCDs leads to reasonable seizure control. However, the success of surgery depends on accurately detecting the epileptogenic lesions during the presurgical evaluations. Therefore, precise detection of FCD lesions is of paramount importance for effective surgical intervention (Radiopaedia, 2024). Among the FCDs, the most common type of epilepsy seen in children is FCD type II, in which most changes occur outside the temporal lobe with predilection for the frontal lobes. FCD type II abnormalities are generally more discernible on brain MRI scans, particularly in fluid-attenuated inversion recovery (FLAIR) MRI imaging, which has high sensitivity in detecting subtle structural abnormalities. Figure 1 displays the FCD type IIb lesion in the left frontal cortex of a 9-year-old female.

Neuroradiologists search for specific imaging markers to identify the focal lesion responsible for a patient’s intractable epilepsy. Commonly, epilepsy detection employs an imaging regimen consisting of FLAIR, T2-weighted, and T1-weighted scans. Moreover, some radiologists might incorporate various imaging techniques such as fluorodeoxyglucose positron emission tomography (FDG-PET), single-photon emission computed tomography (SPECT), and diffusion imaging. Higher field imaging can enhance the identification of crucial imaging features in FCD. However, the manual identification of FCD type II is time-consuming and laborious for clinicians due to very challenging MRI features (Kabat and Król, 2012).

In recent years, deep learning techniques, particularly convolutional neural networks (CNN), have shown significant potential in image classification and segmentation problems since they could learn optimal features automatically (Tiwari et al., 2020). Recent progress in deep-learning-based medical image segmentation has introduced architectures that go beyond classical CNNs. Transformer-based models, such as TransUNet and UNETR, combine self-attention mechanisms with convolutional encoders to capture global context effectively. Hybrid architectures like Swin-UNETR and SegFormer offer efficient hierarchical representation learning while maintaining resolution-specific detail. Lightweight segmentation models, including MobileNetV3-UNet and Fast-SCNN, have also been explored to meet the computational constraints in clinical settings. These models have achieved promising results across modalities like MRI, CT, and PET for segmentation tasks in neuroimaging including brain tumor segmentation, stroke detection, and Alzheimer’s classification, laying the foundation for their application to challenging disorders like FCD. These algorithms enable automated, optimal feature extraction, surpassing the capabilities of traditional manual methods.

While these novel architectures offer competitive performance, they often require substantial manual tuning, architecture customization, and may not generalize well without extensive task-specific adaptation. In contrast, nnU-Net distinguishes itself by being self-configuring, requiring no manual modification for new segmentation problems. This makes nnU-Net not only powerful but also highly practical and reproducible for clinical research.

Adapting AI-assisted algorithms for clinical use faces several hurdles, such as variations in MRI scan quality, inconsistencies in lesion detection results, and particular difficulties with FLAIR MRI images (Gill et al., 2021; Spitzer et al., 2022). Additionally, many slices in NIfTI files lack relevant lesion information, further complicating the detection process.

This study addresses these challenges by proposing an automated FCD type II lesion segmentation approach in 3D FLAIR MRI scans. The main contributions of this work are as follows:

(1) We have used an automated and heuristic slice selection approach that rank each slice by its maximum FLAIR intensity and keeps the five highest-ranked slices per scan, thereby concentrating training on lesion-rich slices while cutting the execution time.
(2) This study seeks to employ the nnU-Net (Isensee et al., 2021) biomedical image segmentation framework, which automatically adapts its configuration including preprocessing, network architecture, training, and post-processing to any new task within the biomedical field.
(3) This study introduces a preprocessing pipeline for 3D FLAIR MRI images, which includes spatial interpolation, skull stripping, intensity normalization, and strategic slice selection to maximize the visibility of epileptogenic lesions. By integrating this pipeline with the nnU-Net framework, we validate its effectiveness on a complex and variable dataset, demonstrating significant improvements particularly in context of FCD type II segmentation.

Apart from the introduction, the remainder of this paper is structured as follows: Section 2 reviews recent advancements and related works in the field. Section 3 details the materials and methods employed in this research. Section 4 presents the results and provides an in-depth discussion. Finally, Section 5 offers concluding remarks and outlines potential directions for future work.

## 2 Recent works

Detecting FCD lesions remains a challenging task, even for experienced radiologists. Studies indicate that approximately 34% of MRI scans from pathologically confirmed FCD cases are reported as MRI-negative, meaning the lesions are not visually apparent on standard scans. However, advancements in automated FCD detection have significantly evolved, incorporating techniques such as image processing, feature extraction, deep learning, statistical analysis, and morphometric analysis (Ganji et al., 2024).

Ganji et al. (2021) extracted morphological and intensity-based features from regions of interest in a cohort of 58 participants. A machine learning-based classification approach was then employed to identify and localise FCD lesions, achieving an impressive sensitivity of 96.7% and specificity of 100%. Similarly, Simozo et al. (2020) developed an automated classification method for FCD lesions using 85 MRI T1-weighted and FLAIR images. They computed 10 cortical features for each subject and utilised a Random Forest classifier, evaluated through Leave-One-Patient-Out (LOPO) cross-validation.

House et al. (2021) introduced a 3D CNN architecture enhanced with autoencoder regularisation to improve FCD detection and segmentation across various FCD subtypes. Their study, conducted on a cohort of 158 patients, yielded a sensitivity of 70.1% and precision of 54.3% for FCD detection. When trained on a dataset that included 100 regular MRI scans, the model achieved a dice score of 0.341.

Wang et al. (2023) proposed a classification model integrating a multiscale receptive field module and a squeeze- and-excitation module to predict FCD type III refractory epilepsy outcomes using T2-weighted FLAIR images. Applied to MRI scans from 260 patients, this model achieved an AUC of 96.22%, sensitivity of 84.47%, and specificity of 97.21%. In another study, Lee et al. (2020) analysed 46 patients with confirmed FCD type II and 35 age and sex-matched healthy controls. Using 3 T multi-contrast MRI imaging, they processed surface-based metrics and employed consensus clustering, an unsupervised learning approach, to identify stable cortical patterns associated with FCD lesions.

Niyas et al. (2021) developed a U-Net-based 3D CNN model that captures inter-slice information from MRI volumes. Their approach incorporated BM3D-based denoising algorithm before training a 3D U-Net model with residual blocks in the encoder. This model achieved a precision of 69.58% and a recall of 61.86% for FCD segmentation. David et al. (2021) performed morphometric analysis on 3D T1-weighted MRI scans to generate 3D morphometric maps. They trained an artificial neural network (ANN) on data from 113 patients with manually segmented FCDs and 362 healthy controls collected from 13 MRI scanners. Their model demonstrated a sensitivity of 87.4%, specificity of 85.4%, and overall accuracy of 85.9% on the testing dataset.

Zheng et al. (2024) introduced a 3D CNN-based model integrating multimodal data using a 3D U-Net backbone. Their study utilised MRI and PET images from 82 patients, achieving a mean sensitivity of 90.3% on the test set. Additional ablation studies were conducted by selectively removing imaging modalities to analyse modality-specific contributions. Feng et al. (2020) introduced a six-layer CNN architecture called NetPos for detecting and segmenting FCD lesions. They utilized the Activation Maximization Convolutional Localization (ACML) algorithm to identify pattern image blocks resembling lesions, which were trained on NetPos. The method was tested on 34 FLAIR MRI images, resulting in a dice coefficient of 52.68.

In addition to these studies, there have been many other attempts to improve the detection and segmentation of FCD using deep learning and machine learning techniques. Despite these advancements, significant opportunities remain to enhance FCD segmentation performance. The study aims to test how well the nnU-Net framework can handle this complicated dataset and automate the segmentation of FCDs.

## 3 Materials and methods

In this section, we outline the nnU-Net architecture, describe the dataset, detail our methodology, discuss the training schedule and parameters, introduce evaluation metrics, and provide implementation details.

### 3.1 Overview of nnU-Net architecture

**Figure 2:**
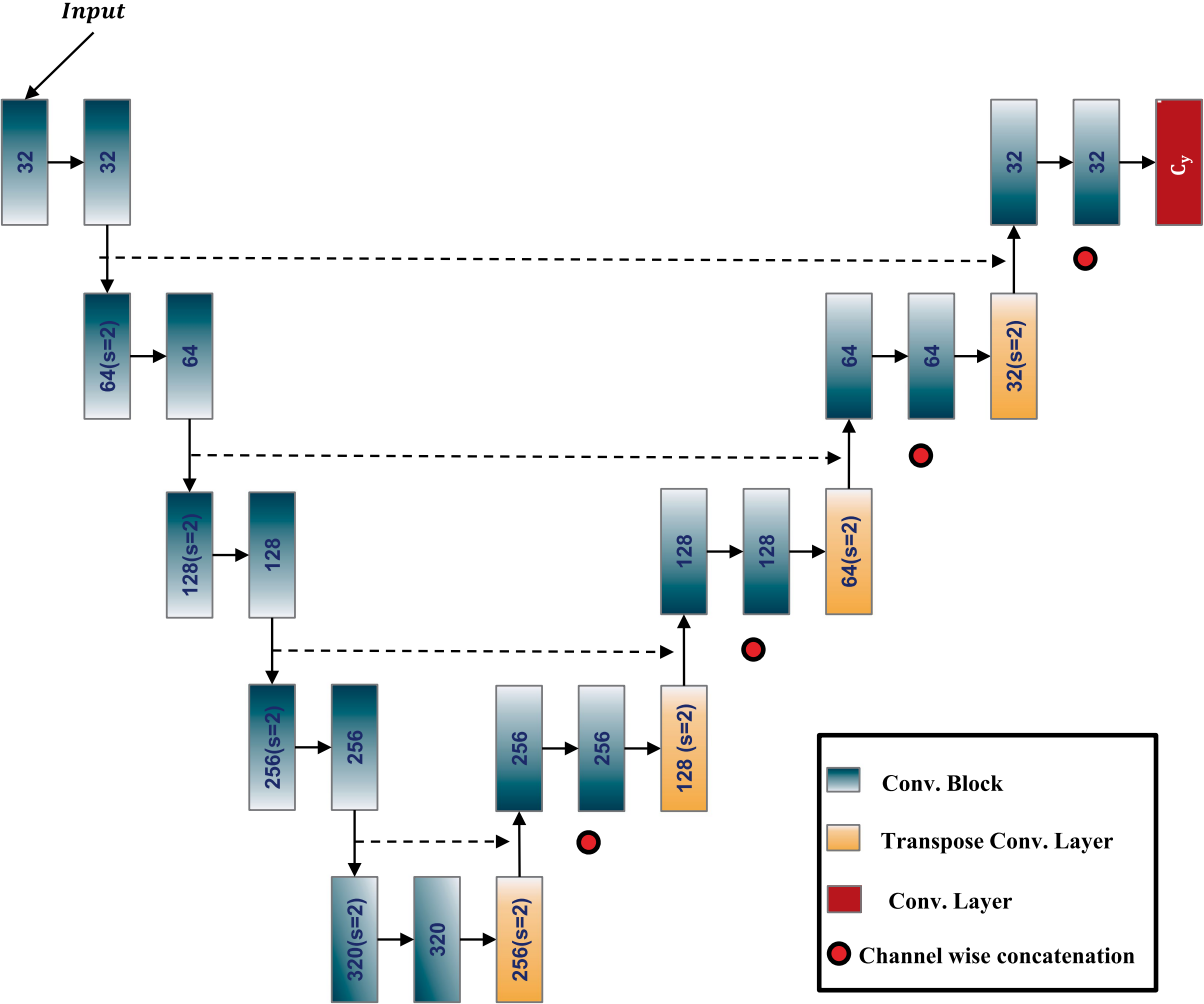
Architecture of 3D ResUNet employed in nnU-Net framework.

The nnU-Net (Isensee et al., 2021), or no-new-Net, is an automated segmentation method that optimizes the U-Net architecture for any given dataset without the need for manual adjustments. It is designed to offer a highly adaptive, robust, and generalizable approach to medical image segmentation tasks. At its core, nnU-Net retains the fundamental structure of the U-Net, a CNN architecture known for its efficacy in biomedical image segmentation. The U-Net architecture features an encoder-decoder structure enhanced by skip connections, allowing the network to capture high-level semantic information while retaining low-level spatial details. The architecture of the adapted 3D ResU-Net in the nnU-Net framework is shown in Figure 2. The nnU-Net architecture adapts itself based on the specific dataset’s properties, including input image size, voxel spacing, and the number of training samples. It systematically tunes various hyperparameters, such as the depth of the network, the number of feature maps, batch size, and patch size, to achieve optimal performance.

The authors of the nnU-Net have validated its performance across 23 public datasets, demonstrating its versatility and effectiveness. This paper extends this evaluation to a novel application, the automatic segmentation of FCD type II lesions on 3D FLAIR MRI images. This exploration aims to not only verify the nnU-Net’s robustness in a new clinical context but also to contribute valuable insights into its potential for enhancing epilepsy diagnosis and treatment planning.

### 3.2 Dataset description

The dataset used in this study is an open-access MRI dataset available on OpenNeuro.org, which was initially collected by the Department of Epileptology at University Hospital Bonn, Germany (Schuch et al., 2023). It comprises high-resolution MRI scans in NIfTI format, including T1-weighted and FLAIR-weighted images from 85 individuals with epilepsy caused by FCD type II and 85 healthy controls. All participants included in the dataset were over 18 years old at the time of data collection. Among the 85 epilepsy patients, 35 (41.2%) were female, and 50 (58.8%) were male. Two experienced neurologists using 3D FLAIR-weighted sequences to ensure precise lesion localisation manually annotate ground truth lesion masks for dysplastic cortical regions. Approximately 62.4% of the identified epileptogenic lesions were located in the frontal lobe of the brain. The University of Bonn ethics committee, ensuring compliance with ethical standards for human research, approves the dataset collection and usage.

### 3.3 Overview of methodology

This study utilises an automated segmentation approach to detect FCD type II lesions, classifying each voxel in the MRI scan as either lesional or non-lesional. The segmentation pipeline is built upon nnU-Net, a self-adapting deep-learning framework designed explicitly for medical image segmentation tasks. The process begins with loading FLAIR-weighted MRI scans in NIfTI format, as dysplastic cortical regions are most prominently visible in FLAIR sequences. Given that the MRI images in the dataset vary in size, spatial interpolation is applied to standardise dimensions across all samples. Following preprocessing steps are performed to enhance image quality before feeding the data into the deep learning model:

- Spatial interpolation: Adjusts voxel spacing to ensure consistency across all scans.
- Skull stripping: Removes non-brain structures to focus solely on the cerebral cortex.
- Intensity normalization: Standardizes voxel intensity values across different MRI scans for improved feature extraction.

After preprocessing, binary masks are generated for regions of interest (ROI). In these masks, lesional pixels are assigned a value of 1, while all other pixels are set to 0, enhancing lesion visibility in epileptic images. Since an MRI volume consists of multiple slices, but only a subset contains maximally visible lesion regions, an automated slice selection strategy is implemented. The pipeline tracks the indices of slices containing lesions and identifies the slice with the highest lesion visibility. The top five ranked slices with the most visible ROIs are then extracted and compiled into a new 3D NIfTI file, ensuring that the input data contains the most relevant information for training.

The 3D full-resolution (3D fullres) architecture of nnU-Net is employed for model training, as it is specifically designed to handle high-resolution 3D datasets. It operates on the full resolution of the input images, unlike the 3D cascade architecture which processes downsampled images first. The 3D fullres nnU-Net typically involves a U-Net-like structure with multiple encoder and decoder layers, incorporating skip connections to preserve spatial information. The input to the model consists of the preprocessed 3D FLAIR MRI scans, along with their corresponding ground truth masks, which experienced neurologists manually segmented. To ensure statistical reliability and mitigate bias, a 5-fold cross-validation strategy is adopted.

**Figure 3:**
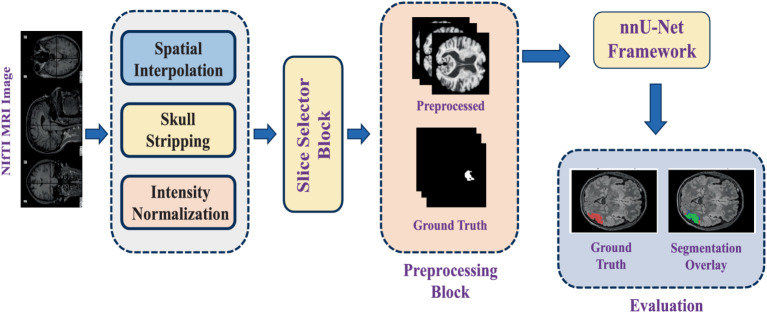
Overview of the methodology.

A detailed workflow diagram illustrating the segmentation process from initial MRI input to final lesion segmentation output is provided in Figure 3.

### 3.4 Training schedule

For the training of our 3D segmentation model, we utilized the nnU-Net framework with a focus on the 3d_fullres configuration. The model was trained for 100 epochs using a batch size of 4, with a learning rate set at 0.01 and an SGD optimizer configured with a momentum of 0.99 and Nesterov acceleration. The training data augmentation pipeline incorporated a variety of transformations, including spatial transformations, Gaussian noise, and intensity adjustments, to enhance model robustness.

### 3.5 Evaluation metrics

To evaluate the segmentation performance, we adopt two complementary metrics: the dice similarity coefficient (DSC) and the pseudo dice score (PDS). These metrics provide insights into the overlap and stability of lesion predictions, particularly relevant in cases of small or imbalanced lesion representations such as FCD type II.

#### 3.5.1 Dice score

Dice similarity coefficient (DSC) (Eelbode et al., 2020) is a standard metric that measures the overlap between the predicted segmentation and the ground truth. The DSC is calculated from Equation 1 as:


(1) $$\mathrm{DSC}=\frac{2\times |A\cap B|}{|A|+|B|}\;$$

where ∣A∣ represents the set of voxels in the predicted segmentation, ∣B∣ represents the set of voxels in the ground truth and ∣A∩B∣ is the number of overlapping voxels, and ∣A∣ and ∣B∣ are the total number of voxels in the predicted and ground truth segmentations, respectively. A DSC score of 1 indicates perfect overlap, while a score of 0 implies no overlap.

#### 3.5.2 Pseudo-dice score

While DSC is effective for many applications, it can become unstable when applied to datasets with highly imbalanced classes or very small lesion volumes.

To address this issue we use the pseudo dice score (PDS). The PDS modifies the DSC by adding a small constant, ${\;}^{“}\varepsilon^{”}$ to both the numerator and the denominator, which helps stabilize the score for small lesion volumes. The formula for the PDS is given by Equation 2 as:


(2) $$\mathrm{PDS}=\frac{2\times (|A\cap B|+\varepsilon )}{|A|+|B|+\varepsilon }\;\;$$

#### 3.5.3 Mean pseudo-dice score

To obtain an overall assessment of the model’s performance across multiple test cases, we compute the Equation 3. This metric is the average of the PDS across all test samples and provides a single value summarizing the segmentation accuracy:


(3) $$\begin{array}{c} \text{Mean}\;\mathrm{PDS}=\frac{1}{N}\sum_{i=1}^{N}{\mathrm{PDS}}_{i} \end{array}$$

#### 3.5.4 Moving average pseudo-dice

In addition to the mean PDS, we calculate the pseudo-dice moving average as given in Equation 4 to observe the performance trend of the segmentation model over time or across iterations. The moving average is handy for monitoring training progress or evaluating model stability. It is calculated as follows:


(4) $$\begin{array}{c} \text{Moving average}\;{\mathrm{PDS}}_{t}=\frac{1}{w}\sum_{i=t-w+1}^{t}{\mathrm{PDS}}_{i} \end{array}$$

where *t* is the current epoch or iteration *w* is the window size (number of past scores to average).

### 3.6 Implementation details

We conducted our experiments on Google Colab Pro, utilizing NVIDIA L4, T4, and A100 Tensor Core GPUs. The experiments were implemented using Python, using the libraries, including PyTorch, NiBabel, NumPy, Pandas, and Matplotlib.

## 4 Results and discussions

In this section, we have presented the performance of the employed model, and further, the statistical analysis has been performed for the experiments done in various folds to ensure that there is no bias in our method. Further, an in-depth discussion is provided of the obtained results in our experiments.

### 4.1 Model performance

**Figure 4:**
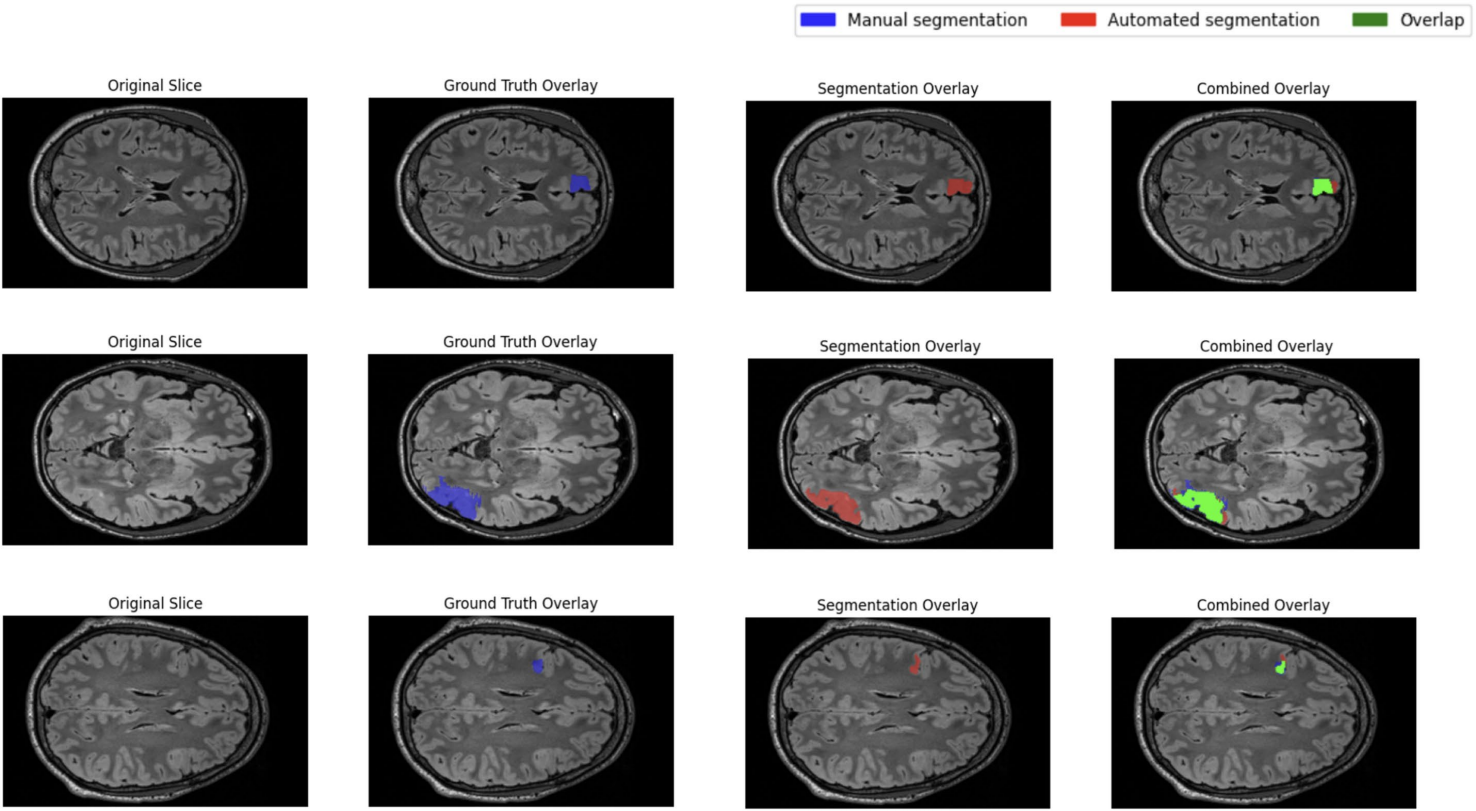
Masks of ground truth, segmentation and overlay for three FCD subjects.

**Figure 5:**
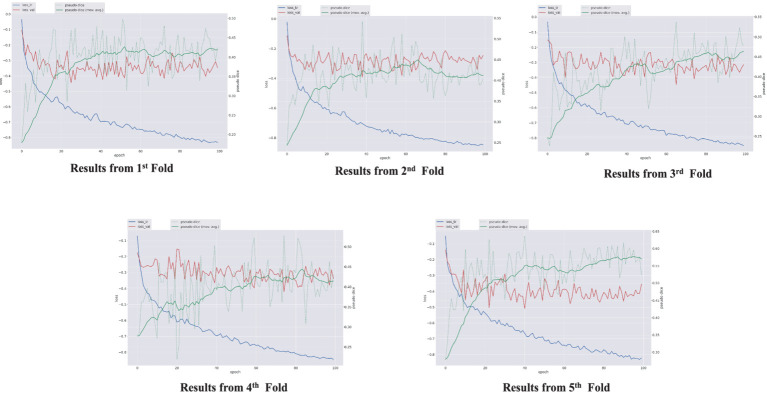
Epochs vs. training and validation loss trend and moving average pseudo dice scores, across five training folds.

The performance of the nnU-Net-based segmentation model was evaluated using 5-fold cross-validation. The predicted segmentation masks were compared against the ground truth labels, and performance metrics were computed accordingly. Table 1 summarizes the results of five different FCD type II segmentation configurations, assessed using internal 5-fold cross-validation. Figure 4 illustrates examples from the three patients from validation dataset, showing the segmented lesion detected by nnU-Net overlaid on the original MRI scan, compared to the ground truth. Figures 5 display the epochs vs. training and validation loss trend and moving average pseudo dice scores, across five training folds. Among the five folds the fold 5 achieved the best lesion segmentation performance, attaining an average PDS of 0.47, with a PDS of 0.52 after the 100th epoch.

**Table 1:** Five-fold cross validation results on FCD dataset.

Fold	Mean pseudo dice	Pseudo dice at 100th epoch
1	0.42	0.42
2	0.29	0.40
3	0.33	0.47
4	0.35	0.42
5	0.47	0.52

### 4.2 Statistical analysis

In this section, we present the results obtained from the 5-fold cross-validation and discuss the statistical analyses conducted to evaluate the performance of our nnU-Net based automatic segmentation model for FCD type II lesions on 3D FLAIR MRI images.

The descriptive statistics for the mean PDS across the five folds are as follows: mean 0.37, standard deviation 0.07, median 0.35. The descriptive statistics for the PDS at the 100th epoch are: mean: 0.45, standard deviation: 0.05, median: 0.42.

#### 4.2.1 Confidence intervals

To further understand the reliability of these scores, 95% confidence intervals were calculated. The confidence interval is (0.28, 0.46) for the mean PDS. This interval signifies 95% confidence that the mean PDS falls within this range. Similarly, for the PDS at the 100th epoch, the confidence interval is (0.39, 0.51), which measures the precision of our estimated mean.

#### 4.2.2 Paired *t*-test

A paired *t*-test was conducted to determine if there is a statistically significant difference between the mean PDS and the PDS at the 100th epoch. The test yielded a *t*-statistic of −3.06 with a *p*-value of 0.04. Since the *p*-value is less than 0.05, we reject the null hypothesis and conclude that there is a statistically significant difference between the two sets of scores. This result suggests that the model’s performance improved over the course of training, as evidenced by the higher PDS at the 100th epoch compared to the mean PDS.

## 5 Discussion

The results indicate that the nnU-Net-based model achieved a mean PDS of 0.37 across the five folds, with a standard deviation of 0.07. The PDS at the 100th epoch improved, with a mean of 0.45 and a standard deviation of 0.05. The confidence intervals provide further assurance of the reliability of these estimates.

Given the differences in dataset sizes and study subjects, directly comparing results from different methods may not provide a fair assessment. However, to understand the performance of our approach relative to other FCD segmentation studies, we conducted a comparison. The results show that the nnU-Net method we applied produces results that are on par with those of similar studies. Table 2 shows the comparison of FCD segmentation performance across the different datasets.

**Table 2:** Comparison of FCD segmentation performance across different methods.

Dataset/Modality	Architecture	Dice score	Reference
MRI (201 FCD subjects)	Encoder decoder based CNN	0.341	House et al. (2021)
EPISURG dataset	MATPR-UNet	0.42 ± 0.08	Zhang et al. (2024)
18 FLAIR negative MRI scans	Net-Pos (CNN)	0.5268	Feng et al. (2020)
3D FLAIR MRI (85 subjects)	nnU-Net	0.52 (best)	This study
		0.45 (mean)	

The paired *t*-test results highlight a significant improvement in the model’s performance from the mean PDS to the scores at the 100th epoch. This improvement is critical for effectively segmenting FCD type II lesions, as it demonstrates the model’s ability to learn and adapt during training.

Qualitative inspection of three validation-set patients selected for their distinctly different lesion volumes and cortical locations shows that the network generalises well across this spectrum of presentations. In every case, the predicted mask aligns closely with the manual ground truth, regardless of lesion size or cortical lobe, and does so while avoiding spurious activations in healthy cortex. This consistency supports the model’s robustness and its potential clinical utility for reliably flagging FCD II lesions in heterogeneous real-world data.

The detection and segmentation of FCD is still a difficult issue with potential for dice score accuracy improvement, even with noteworthy advances in computer vision and segmentation architectures. It is believed that the segmentation performance will continue to improve as more data is acquired and training epochs are increased. Additional data will help the model better grasp the variability in FCD type II lesions, which will produce segmentation findings that are more reliable and accurate. The model’s performance can be improved and its weights further refined by extending the training period to further epochs. To attain even greater segmentation accuracy, future research will concentrate on these factors.

## 6 Conclusion

This study confirmed the effectiveness of the 3D full-resolution nnU-Net in reliably segmenting FCD lesions from the 3D FLAIR MRI images. The models achieved a maximum PDS of 0.52 on the validation dataset. As the FCD lesions are very complex and frequently display subtle and heterogeneous traits, it is imperative to improve the segmentation models’ capacity to capture these differences appropriately. As a result, in the future, more advanced segmentation methods can be used to identify epileptogenic lesions in 3D FLAIR MRI images and other MRI modalities and the fusion of multimodal data can be adopted for the accurate segmentation.

To enhance feature extraction and lesion localization, future studies should create more sophisticated segmentation methods including transformer-based models, attention-based U-Nets, and hybrid architectures. Furthermore, combining FLAIR, T1-weighted, T2-weighted, PET, and diffusion MRI images with multimodal data fusion may offer a more thorough depiction of epileptogenic lesions, improving segmentation performance. In order to improve interpretability and give doctors clear insights into the model’s decision-making process, explainable AI (XAI) approaches such as Grad-CAM, SHAP, and LIME are being integrated. This is another critical area for improvement.

Furthermore, future research should concentrate on creating lightweight 3D segmentation architectures that can function effectively with constrained computational resources while preserving high segmentation accuracy, considering the hardware limitations in actual clinical settings. Model generalizability could be further improved by extending training strategies by expanding dataset size, refining cross-validation procedures, and investigating semi-supervised or self-supervised learning methodologies. By addressing these issues and implementing these developments, automated FCD lesion segmentation will be enhanced, ultimately resulting in more accurate epilepsy diagnosis and better treatment planning. Neuroradiologists may be able to identify better epileptogenic lesions with the help of AI-driven segmentation techniques integrated into clinical processes, improving patient care and surgical results.

## Data Availability

The original contributions presented in the study are included in the article/supplementary material, further inquiries can be directed to the corresponding author.
